# Multi-feature fusion learning for Alzheimer's disease prediction using EEG signals in resting state

**DOI:** 10.3389/fnins.2023.1272834

**Published:** 2023-09-25

**Authors:** Yonglin Chen, Huabin Wang, Dailei Zhang, Liping Zhang, Liang Tao

**Affiliations:** ^1^Anhui Provincial Key Laboratory of Multimodal Cognitive Computation, Hefei, China; ^2^School of Computer Science and Technology, Anhui University, Hefei, China; ^3^Faculty of Engineering, Malaysia School of Engineering, Monash University, Kuala Lumpur, Malaysia; ^4^Hospital Information Management Center, The Second Hospital of Anhui Medical University, Hefei, China

**Keywords:** Alzheimer's disease (AD), electroencephalography (EEG) signal, dual-branch feature fusion, multiattention mechanism, two-factor diagnosis

## Abstract

**Introduction:**

Diagnosing Alzheimer's disease (AD) lesions via visual examination of Electroencephalography (EEG) signals poses a considerable challenge. This has prompted the exploration of deep learning techniques, such as Convolutional Neural Networks (CNNs) and Visual Transformers (ViTs), for AD prediction. However, the classification performance of CNN-based methods has often been deemed inadequate. This is primarily attributed to CNNs struggling with extracting meaningful lesion signals from the complex and noisy EEG data.

**Methods:**

In contrast, ViTs have demonstrated proficiency in capturing global signal patterns. In light of these observations, we propose a novel approach to enhance AD risk assessment. Our proposition involves a hybrid architecture, merging the strengths of CNNs and ViTs to compensate for their respective feature extraction limitations. Our proposed Dual-Branch Feature Fusion Network (DBN) leverages both CNN and ViT components to acquire texture features and global semantic information from EEG signals. These elements are pivotal in capturing dynamic electrical signal changes in the cerebral cortex. Additionally, we introduce Spatial Attention (SA) and Channel Attention (CA) blocks within the network architecture. These attention mechanisms bolster the model's capacity to discern abnormal EEG signal patterns from the amalgamated features. To make well-informed predictions, we employ a two-factor decision-making mechanism. Specifically, we conduct correlation analysis on predicted EEG signals from the same subject to establish consistency.

**Results:**

This is then combined with results from the Clinical Neuropsychological Scale (MMSE) assessment to comprehensively evaluate the subject's susceptibility to AD. Our experimental validation on the publicly available OpenNeuro database underscores the efficacy of our approach. Notably, our proposed method attains an impressive 80.23% classification accuracy in distinguishing between AD, Frontotemporal dementia (FTD), and Normal Control (NC) subjects.

**Discussion:**

This outcome outperforms prevailing state-of-the-art methodologies in EEG-based AD prediction. Furthermore, our methodology enables the visualization of salient regions within pathological images, providing invaluable insights for interpreting and analyzing AD predictions.

## 1. Introduction

Alzheimer's disease (AD) casts a haunting shadow over the lives of those affected, being a neurodegenerative disorder that can eventually lead to dementia (Hecht et al., 2018; Kumar et al., 2018). The diagnostic process for AD involves a plethora of techniques aimed at unraveling the complex structure and function of the nervous system (Taly et al., 2009). In this realm of exploration, electroencephalography (EEG) emerges as a captivating tool, offering profound insights into the enigmatic brain wave signaling abnormalities in AD patients (Wang et al., 2018). By deciphering the cerebral cortex signaling changes, EEG becomes a beacon of hope for early detection and intervention before the onset of distressing symptoms, providing crucial time for AD patients' delay and rehabilitation.

Resting-state EEG signals become the portal through which AD's secrets are unveiled. Clinical studies have uncovered distinct frequency bands in EEG signals that differentiate AD disease progression. The haunting delta and theta waves surge, while the mesmerizing alpha power gracefully diminishes in EEG signals from individuals at different disease stages. However, acquiring reliable EEG signals is challenging, as they may be influenced by human factors and environmental noise, concealing the celestial truth (Gouw et al., 2011). The complex and nonlinear nature of EEG signals demands efficient feature extraction and screening methods to reveal the elusive critical AD lesion signals.

In this quest, two factions emerge: powerful machine learning methods and enigmatic deep learning methods. The former, utilizing decision trees, multilayer perceptrons, nearest neighbors, and the indomitable support vector machines (SVMs), identify various disorders, including AD, schizophrenia, and epilepsy. Among them, Trambaiolli et al. (2011) harnessed SVM to discern AD patients with unprecedented speed and accuracy. The latter faction, steeped in mystery, leverages the magic of deep learning to foretell the future of disease prediction. Yu et al. (2019) extracted topological features of functional brain networks and prophesied AD diseases using a mystical supervised network. Li et al. (2021) ventured into latent factors using a variational self-encoder, elevating the accuracy of AD prediction.

The Transformer stands as an illustrious champion in the arcane realm of deep learning models. It gracefully combats the interference of high-dimensional data noise on EEG signals, revealing the elusive secrets within the brain. Yet, variations like ViTs (Wang et al., 2021), which unveil global representations, stumble in capturing the essence of local details essential for unearthing the critical AD lesion signals in the mystical frequencies (Wu et al., 2021). The grand union of ViTs and CNNs harmonizes their strengths and dissipates their weaknesses, yielding unprecedented power in AD prediction through medical signal image classification.

Fouladi et al. (2022) introduced two distinct deep learning architectures—Convolutional Neural Networks (CNN) and convolutional autoencoder neural networks—for the classification of subjects into AD, Mild Cognitive Impairment (MCI), and NC categories using scalp EEG recordings. Notably, the modified convolutional network demonstrated an average accuracy of 92%, while the convolutional autoencoder network achieved 89%. In a separate contribution, Miltiadous et al. (2023a) presented an innovative approach to AD EEG classification utilizing a Dual-Input Convolution Encoder Network (DICE-net). Their methodology involved denoising EEG data, extracting Band Power and Coherence features, and inputting them into the DICE-net architecture, comprising Convolutional, Transformer Encoder, and Feed-Forward layers. Impressively, DICE-net exhibited promising outcomes, achieving an accuracy of 83.28% in the AD-CN problem using Leave-One-Subject-Out validation. This performance surpassed several baseline models, highlighting the method's efficacy and notable generalization capability. In the domain of EEG emotion recognition, Guo et al. (2022) presented a Transformer-based methodology for classifying EEG data based on emotional states. Their approach achieved an accuracy of 83.03% in a three-class problem, outperforming the majority of published methods within the same database. Such investigations underscore the Transformer encoder's efficacy within EEG tasks and underline the imperative to explore its application in the realm of neurodegenerative EEG classification, including AD and other forms of dementia.

Nevertheless, deep learning methods, while magnificent in extracting EEG signals' AD lesion features, remain fragments of the comprehensive analysis required for evaluating the subject's risk of AD. To complete this mystical puzzle, clinical neuropsychological assessments, such as the renowned MMSE (Chapman et al., 2016), interweave their threads. In this fusion, AD prediction results from EEG signals intertwine with clinical neuropsychological assessment results, yielding an all-encompassing prophecy of the subject's risk of disease.

In conclusion, the quest to predict AD through EEG signals reveals gaps in understanding. Deep learning networks, while superior to traditional methods, struggle to capture both global signal features over time bands and local texture signal features over frequency bands. Noise veils the true essence of EEG feature signals, necessitating screening to elevate the mystical lesion information. To fully realize the prophecy of AD, one must combine EEG signal prediction with clinical psychology scale assessment results, embracing the full spectrum of the subject's destiny and unlocking timely intervention and care for those touched by this mysterious malady. Our contributions in this study are highlighted as follows:

**Contribution 1:** We introduce a novel feature level fusion strategy that combines local textural features (EEG signals generated by 19 channels) extracted by CNN and global features (EEG signals in different frequency bands) extracted by ViT. This fusion approach enhances the discriminative power of the features, resulting in superior overall classification accuracy for EEG signals compared to the state-of-the-art (SOTA) methods.

**Contribution 2:** We construct an EEG signal feature attention module, comprising spatial attention and channel attention blocks, which performs feature screening of the fused features. This attention mechanism expands the EEG signal feature information relevant to AD pathogenesis while effectively suppressing noise information. Our ablation experiments demonstrate that the feature attention module significantly improves the network's ability to capture AD-related feature information, leading to enhanced classification accuracy.

**Contribution 3:** We propose a more rational two-factor assessment mechanism for clinical AD risk, integrating Factor 1 (prediction results of our proposed AD prediction method based on EEG signals) and Factor 2 (results of the Clinical Neuropsychological Assessment Scale scores, i.e., MMSE scores). Utilizing correlation analysis, we assess the reasonability of Factor 1 by analyzing the correlation between predicted results of different signal segments from the subject's EEG signals. We then combine the results of Factor 1 with the MMSE scores to predict the subject's risk of AD. Our two-factor AD diagnosis achieves a remarkable 80.23% classification accuracy in the AD vs. FTD vs. NC classification task, effectively reducing the unreliability of diagnostic results associated with single-method predictions.

The structure of the paper is organized as follows: In Section 2, we present a comprehensive review of related works in AD diagnosis using EEG signals. Section 3 elaborates on our proposed method, encompassing feature fusion, multi-attention module, and the two-factor decision-making mechanism. The dataset used in our experiments is detailed in Section 4, along with the presentation of experimental results. In Section 5, we thoroughly evaluate the effectiveness of our proposed method and provide an insightful discussion of the obtained results. Finally, Section 6 concludes the paper by summarizing the contributions and findings of this study.

## 2. Related work

Over the past few decades, numerous AD prediction methods based on EEG signals have been proposed. In the literature, several comprehensive review papers have covered this topic, such as Roy et al. (2019) and Merlin Praveena et al. (2022). In this section, our focus will be on reviewing representative studies from three distinct approaches: (1) traditional machine learning-based AD prediction methods utilizing EEG signals; (2) state-of-the-art deep learning-based AD prediction methods employing EEG signals; and (3) AD prediction methods leveraging multimodal data.

### 2.1. Traditional AD prediction methods

Traditional machine learning methods have been widely employed for disease prediction using EEG signals, particularly for Alzheimer's disease (AD). In one study by Neto et al. (2016), frequency and time domain features of AD and normal control (NC) classes were extracted from EEG data using SVM. The trained model demonstrated an accuracy of 67% in classifying AD.

Another investigation by Trambaiolli et al. (2017) focused on identifying the frequency waves most sensitive to AD disease, aiming to filter the most effective frequency bands for model training. Their experimental findings revealed that alpha and theta waves were most relevant to AD onset. Upon inputting these waves into an SVM model for iterative training, the accuracy in distinguishing between AD and NC classes improved significantly, reaching 71.18%. This observation highlights the importance of selecting appropriate frequency bands to enhance the predictive classification performance of the classifiers.

Furthermore, Kashefpoor et al. (2016) explored the pathogenic relationship between NC and FTD using EEG signals. They adopted a correlation analysis algorithm to extract the electrode channel signal with the highest correlation. Subsequently, this signal was utilized as input for a Neuro-Fuzzy k-Nearest Neighbor Classifier (NF-KNN) during iterative training. As a result, the FTD prediction accuracy on the test set reached 58.89%.

Despite the successes in extracting AD onset signals from EEG data through machine learning techniques, it is essential to acknowledge the reliance on cumbersome pre-processing methods. This aspect poses a significant challenge for early clinical AD screening, where accuracy and environmental adaptability are critical factors to consider.

### 2.2. Deep learning for prediction methods

Deep learning has gained widespread recognition for its ability to extract complex information from various types of data. In recent years, there has been an increasing focus on leveraging deep learning techniques to process and analyze EEG signals related to AD onset. It is believed that deep learning can effectively capture AD onset signals present in EEG data.

One notable study conducted by Duan et al. (2020) utilized the Fast Fourier Transform (FFT) algorithm to extract spectral power information from EEG signals. This unpreprocessed spectral information was then directly input into a CNN for iterative training, resulting in an impressive 79% accuracy in differentiating between AD and NC subjects.

Similarly, Rad et al. (2021) performed EEG experiments on 63 AD subjects, 63 subjects with MCI, and 63 NC subjects. They innovatively transformed the EEG signals into two-dimensional grayscale images, incorporating AD lesion features. These grayscale images were then used as inputs for a CNN model to predict and classify AD subjects, achieving an accuracy of 73.33%. In another study, Amini et al. (2018) focused on the classification task of AD and NC classes. They extracted both time-domain and frequency-domain information from EEG signals, which were then fed into a CNN with pre-trained weights. The final classification accuracy on the test set was an encouraging 82.30%. Notably, these deep learning algorithms demonstrated higher accuracy and simpler preprocessing steps compared to traditional machine learning approaches.

Despite the promising results achieved by these advanced algorithms, it is essential to acknowledge the limitations of EEG signals. EEG signals only provide insights into the changes in electrical brain activity and may carry the risk of misdiagnosis in clinical assessments of AD. Therefore, while deep learning methods have shown remarkable potential, further research and integration of complementary diagnostic information are crucial to enhance the accuracy and reliability of AD prediction in clinical settings.

### 2.3. Multimodal fusion schemes for prediction methods

In the realm of AD prediction, relying solely on a single piece of information, such as EEG signals, may not offer sufficient reliability for clinical assessments. As a result, Chen et al. (2022) and Sharma et al. (2022) researcher have increasingly turned to multimodal fusion strategies to enhance predictive accuracy.

For instance, You et al. (2020) proposed a cascade neural network approach that incorporates both EEG signals and human gait characteristics for classifying AD, MCI, and NC subjects. Their model incorporated a spatial attention mechanism, effectively extracting spatial features from the EEG signals and gait data. This model achieved a classification accuracy of 79.70% on the test set.

Similarly, Ullah et al. (2018) presented a bimodal AD prediction model that integrated time-frequency features from EEG signals and PET image features related to AD lesions. The EEG signals underwent wavelet Fourier transform for time-frequency feature extraction, while the PET features were obtained through dimensionality reduction processing. An improved CNN was iteratively trained using these bimodal features, resulting in a 77.10% accuracy on the test set, with model stability improved by employing five-fold cross-validation.

Another approach was taken by Rad et al. (2021), who augmented the data with three distinct modal features: frequency features, zero-domain signal features, and triggering event signal features. These three features were then input into a multi-channel deep convolutional neural network, achieving a noteworthy 75.50% accuracy in AD classification.

The success of these methods can be attributed to their ability to leverage multiple information sources for AD prediction. In this study, we draw inspiration from these approaches and propose a bimodal joint AD prediction model aimed at assessing the risk of AD prevalence. Our model analyzes the consistency between the predicted results of EEG signals from subjects and combines them with the outcomes of Clinical Psychology Scale assessments to arrive at a final prediction for the risk of developing AD in the subjects.

By integrating information from multiple modalities, our proposed model seeks to improve the accuracy and robustness of AD risk prediction. Through this investigation, we aim to contribute to the growing body of research that explores the potential of multimodal fusion strategies for enhancing AD prediction methods and, ultimately, advancing early detection and intervention in AD-related conditions.

## 3. Methods

**Figure 1:**
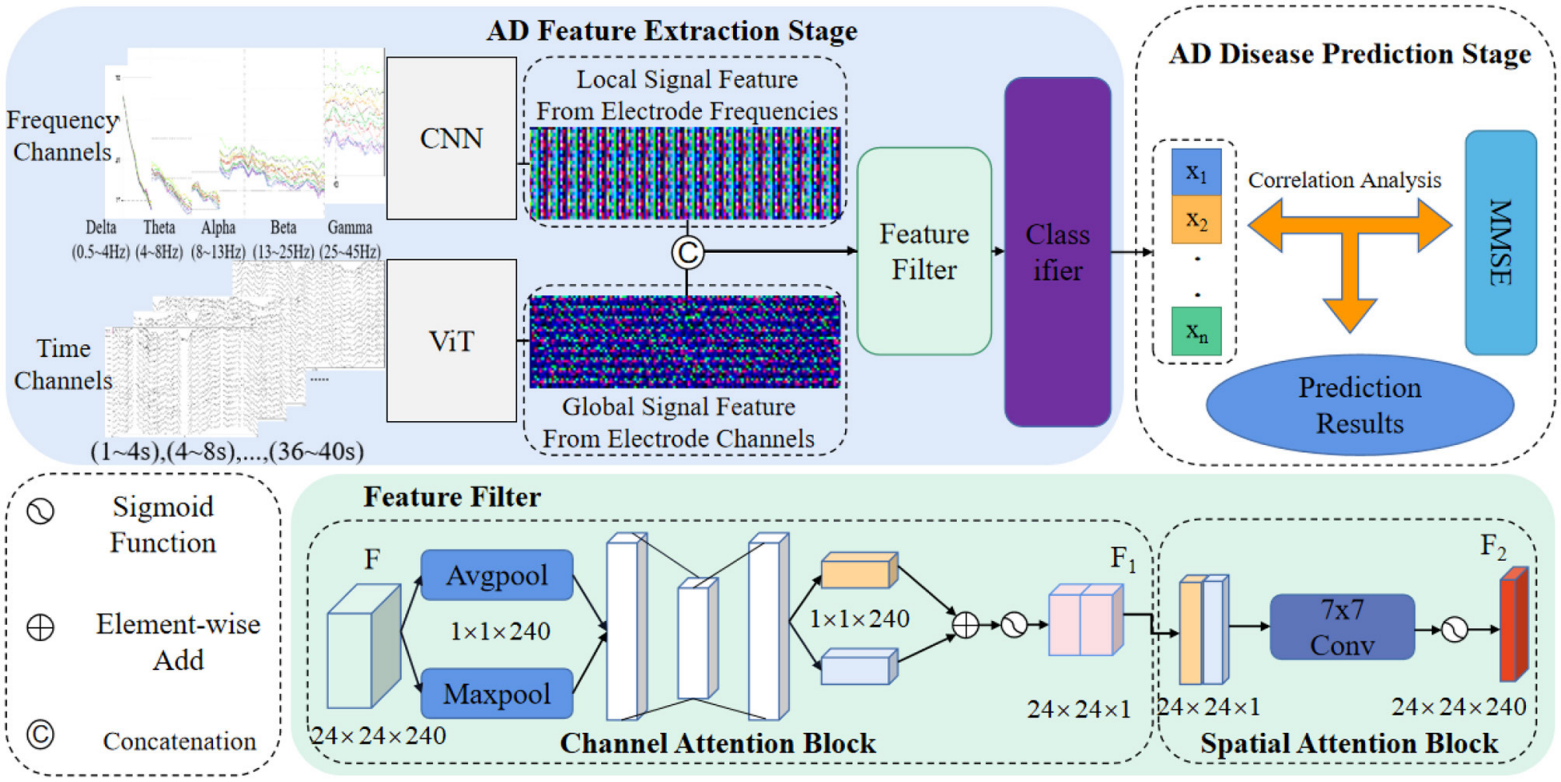
AD prediction pipeline based on the feature fusion strategy using EEG signals.

In this section, we present a comprehensive theoretical analysis of our novel AD prediction pipeline, which is designed to leverage EEG signals. The pipeline incorporates feature fusion strategies derived from ViT and CNN architectures, facilitating the capture of crucial signal information across diverse domains. Specifically, we integrate feature-level fusion strategies, hybrid attention modules, and two-factor decision mechanisms into our proposed method. The visual representation of our approach is depicted in Figure 1. Through this theoretical analysis, we aim to elucidate the underlying principles and mechanisms that underpin the effectiveness and innovation of our AD prediction pipeline, thereby contributing to the advancement of AD diagnostic methodologies based on EEG signals.

As depicted in Figure 1, our method's AD feature extraction phase involves the fusion of detailed features extracted by the CNN from the frequency domain with the global features acquired by the ViT from the time domain. This fusion process yields a comprehensive representation of the EEG signals, capturing both local and global signal characteristics. Subsequently, the fused features are concatenated and directed into a feature filtering module, where we leverage channel attention and spatial attention modules to suppress redundant information and emphasize the salient lesion-related signal features. This selective feature screening enhances the discriminative power of our method for AD prediction.

Following the feature filtering process, the filtered feature information is input into a classifier to generate classification results. These results are then forwarded to our proposed two-factor decision-making mechanism, which forms the core of the AD disease prediction stage. Specifically, a correlation analysis is performed based on the classification results obtained from the EEG signals. This correlation analysis aims to establish the risk prediction outcomes pertaining to the subject's health status, namely, whether the individual is affected by AD or not. The comprehensive process for predicting AD disorders based on EEG signals is concisely outlined in Algorithm 1.

**Algorithm 1:** The Pseudo-code for feature extraction process based on EEG signals.

**Input** *D*_*p*_: training dataset; *p* is number of the training batch; Feature map $F_{vit}^{p}\in {ℜ}^{H\times W\times C}$ with *H* × *W* × *C* dimensions from the ViT; Feature map $F_{cnn}^{p}\in {ℜ}^{H_{i}\times W_{i}\times C_{i}}$ with *H*_*i*_ × *W*_*i*_ × *C*_*i*_ dimensions from the CNN; *i* the final layer number.**Output** *Classifier*(): predicted class for $F_{fuse}^{p}$.
1: for *p* = 1 to *n* **do** do
2: Calculate global feature in EEG electrode signal $F_{vit}^{p}$ based on Equations (2), (3), and (4);
3: Capture the characteristics of the lesion in the EEG frequency band signal $F_{cnn}^{p}$ from CNN ;
4: Calculate $F_{fuse}^{p}$ normalized based on Equation (1);
5: Calculate *F*_1_ = *M*_*s*_(*F*_*f*_*use*) based on Equation (6);
6: Calculate *F*_2_ = *M*_*s*_(*F*_1_) based on Equation (7);
7: end **for**
8: Return *Classifer*((*F*_2_) = *FC*(σ(*FC*(*F*_2_))).

### 3.1. Feature level fusion strategy and loss functions

In recent years, numerous AD prediction methods have utilized EEG signals, yielding favorable outcomes through feature fusion strategies. Among these strategies, the most commonly employed approach involves simple fusion based on elementary operations. In this study, we propose a feature-level fusion strategy that combines the strengths of ViT and CNN through straightforward element-level connectivity.

Specifically, the ViT offers the capability to extract essential features from a multitude of signal waves generated by various electrodes in a subject. This is particularly significant as these waves often encompass a substantial degree of noise, and the inherent feature information can prove elusive to capture. ViT, however, adeptly captures the holistic feature information of the signal wave, presenting a global semantic understanding. In contrast, the CNN excels in extracting local features from frequency signals across diverse frequency bands, encapsulating texture features inherent in the waveforms. A prominent example is the distinctive frequency waveform variations observed in AD patients, typified by reduced alpha power and heightened theta power. The fusion of the complementary attributes of ViT and CNN yields a more comprehensive and discerning representation of EEG data. Notably, this amalgamation facilitates the incorporation of both global and local characteristics, enhancing the potential for discriminative insights. Furthermore, the model's training employs an iterative approach with the cross-entropy loss function. This strategic utilization enhances the model's capacity to capture AD lesion-related information, ultimately contributing to an overall improvement in classification performance.

#### 3.1.1. Feature level fusion strategy

The model follows the outlined structure. Initially, two parallel blocks are established, each receiving input denoted as $X_{i}\in {ℜ}^{B\times H\times W\times C}$, with B representing the batch size. The dimensions of the features extracted by the CNN (19, 128) and the ViT (19, 128) are aligned with these blocks. These dimensions correspond to one block each for CNN and ViT. The fusion of CNN and ViT features is executed employing parameters (19, 256). Subsequently, a concatenation layer is employed, followed by the application of a Feed-Forward Network (FFN). This FFN is pivotal in determining the classification of the input (19, 3).

Figure 1 shows that the feature fusion strategy is two different networks learning characteristic feature information from the EEG signal and fusing them at the channel scale to capture the AD lesion information contained in both feature information. Specifically, the source of the fused features is the last layer of the feature matrices of ViT ($F_{vit}\in {ℜ}^{H_{i}\times W_{i}\times C_{i}}$) and CNN ($F_{cnn}\in {ℜ}^{H_{i}\times W_{i}\times C_{i}}$), and the two matrix features are fused by matrix splicing. The fused feature is $F_{fuse}\in {ℜ}^{H_{i}\times W_{i}\times \left(C_{vit}^{i}+C_{cnn}^{i}\right)}$.


(1) $$\begin{array}{l} F_{flf}^{i}=Concat\left(F_{vit}^{i},F_{cnn}^{i}\right) \end{array}$$

where *Concat*() is the concatenation operation.

#### 3.1.2. Vision Transformer (ViT)

ViT is an extension of the Transformer architecture, comprising a patch embedding, a set of computation blocks, and an encoder. The patch embedding step involves dividing the input image into smaller fixed-size patches. The encoder leverages these computation blocks to process the patch embeddings, capturing the global contextual information and enabling ViT to excel at processing and understanding images on a holistic level.

**Figure 2:**
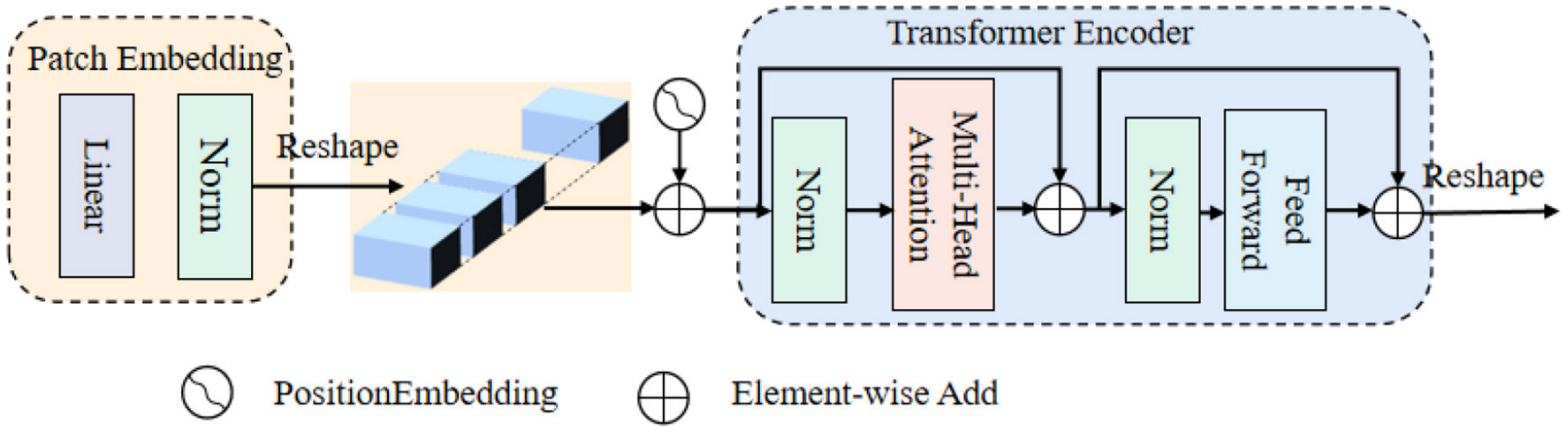
The Vision Transformer block.

Figure 2 shows that: the beginning of stage *i*, let us evenly divide the input feature map $F_{i}\in {ℜ}^{H_{i}\times W_{i}\times C_{i}}$ into $\frac{H_{i}\times W_{i}}{d_{i}^{2}}$ patches, and the patch is flattened and projected to a *C*_*i*_-dimensional embedding. After the linear projection, the shape of the embedded patch is F (i.e., $\frac{H_{i}}{d_{i}}\times \frac{W_{i}}{d_{i}}\times C_{i}$), where the height and width are *d*_*i*_ times smaller than the input, the patch size of the *i*-th stage as *d*_*i*_.

Transformer encoder, which is composed of an multi-head attention (MHA) layer and a feed-forward layer. Here, the MHA receives a query *q*, a key *k*, and a value *v* as input. The formula can be expressed as follows:


(2) $$\begin{array}{l} MHA\left(q,k,v\right)=Concat\left(head_{0},\cdots \;,head_{N_{i}}\right)W^{0} \end{array}$$

(3) $$\begin{array}{l} head_{j}=Attention\left(QW_{j}^{q},KW_{j}^{k},VW_{j}^{v}\right) \end{array}$$

where *Concat*() is the concatenation operation. $W_{j}^{q},W_{j}^{k},W_{j}^{v}\in {ℜ}^{C_{i}\times d_{head}}$, are linear projection parameters. *Attention*() is calculated as:


(4) $$\begin{array}{l} Attention\left(q,k,v\right)=Softmax\left(\frac{qk^{T}}{\sqrt{d_{head}}}v\right) \end{array}$$

#### 3.1.3. Loss functions

We employ the cross-entropy loss function for iterative model optimization. Specifically, we utilize the CNN to capture fine-grained AD onset frequency information in the frequency domain and the ViT to extract brainwave signals in the time domain. The model is optimized through iteratively updating the cross-entropy loss with the corresponding labels, thus facilitating efficient discrimination between AD, FTD, and NC subjects. Through this iterative optimization process, our model is trained to accurately predict AD diseases by effectively leveraging the distinctive features captured from both the frequency and time domains of EEG signals. The loss formula is expressed as follows:


(5) $$L_{CE}=\{\begin{array}{c} \frac{1}{N}\sum_{i}-\left[{\hat{y}}_{i}\cdot \log(p_{i})+(1-{\hat{y}}_{i})\cdot \log(1-p_{i})\right]\\ \;-\frac{1}{N}\sum_{i}\sum_{c=1}^{M}y_{ic}\log(p_{ic}) \end{array}$$

where *N* is the number of samples, *M* is the disease classification, *y*_*i*_ is the label of subject *i*, which is 1 if the prediction is consistent with the label and 0 otherwise, *p*_*i*_ is the probability that subject *i* is predicted by the model to be consistent with the label, *y*_*ic*_ is the sign function (0 or 1), and *p*_*ic*_ is the outcome of subject *i* being predicted by the model.

### 3.2. Feature filtering module

Despite the feature extraction process using ViT and CNN, the extracted EEG signal feature information may still contain substantial noise and redundancy, which can adversely affect the classification performance and operational efficiency of the prediction model. To address this issue, this paper introduces a feature screening module, enabling the network to focus more selectively on significant features in the information while suppressing noise and redundant elements, as illustrated in Figure 1 (Feature filter).

The constructed EEG signal feature screening attention module comprises a channel attention module and a spatial attention module. Taking the fused EEG signal feature map *F*_*f*_*use* as an example (Figure 1), the feature *F*_*f*_*use* undergoes maximum pooling, and global pooling, resulting in two 1×1×64 channel vectors. These vectors are then fed into a fully connected layer to obtain activated weighting coefficients *M*_*s*_. These coefficients are then multiplied with the fused feature *F*_*f*_*use*, generating a new feature *F*_1_.

Subsequently, *F*_1_ undergoes an average pooling layer and a maximum pooling layer, producing two 40 × 48 × 1 feature vectors. These vectors are concatenated and passed through a 7 × 7 convolutional layer, generating new weighting coefficients *M*_*s*_. The resulting coefficients are then multiplied with *F*_1_, yielding the new feature *F*_2_. The mathematical formula for this process is as follows:


(6) $$\begin{array}{l} M_{c}\left(F_{f}use\right)=\sigma \left(MLP\left(AvgPool\left(F_{f}use\right)\right)+MLP\left(MaxPool\left(F_{f}use\right)\right)\right) \end{array}$$

(7) $$\begin{array}{l} M_{s}\left(F_{1}\right)=\sigma \left(f^{7\times 7}\left(\left[AvgPool\left(M_{c}\left(F_{1}\right)\right),MaxPool\left(M_{c}\left(F_{1}\right)\right)\right]\right)\right) \end{array}$$

where *MLP* is the two-layer perception module, *AvgPool* is the average pooling module, *MaxPool* is the maximum pooling module, *F*_*f*_*use* is the input fusion feature vector, and *f* is the convolution module.

Through this feature screening attention mechanism, our approach effectively enhances the model's ability to focus on informative features while attenuating noise and redundancy, contributing to improved classification performance and overall prediction model efficiency. The incorporation of the channel and spatial attention modules further ensures the selection of crucial features, promoting the accuracy and reliability of the AD prediction model.

### 3.3. Two-factor decision-making mechanism

In this study, we employ correlation analysis, an analytical algorithm widely used to explore the relationships between different variables. The correlation coefficient quantifies the strength of the linear relationship between variables under investigation. In our approach, the proposed network performs EEG signal classification for prediction, where NC is 0, FTD is 1, and AD is 2. To ensure the credibility of the EEG signal prediction results, we compare them with the clinical assessment results obtained from the MMSE. When the EEG signal prediction aligns with the MMSE clinical assessment result, it is considered reliable; otherwise, it is deemed less credible.

To quantitatively assess the correlation between the predicted EEG signal results and the MMSE clinical psychology scale, we utilize Pearson's correlation coefficient, a statistical measure that helps evaluate the degree of correlation between these variables. This analysis provides valuable insights into the reliability of our prediction model.

By integrating EEG signal predictions with clinical neuropsychological assessments and incorporating correlation analysis, our two-factor decision-making mechanism enhances the accuracy and confidence of clinical AD diagnosis. This approach represents a significant advancement toward a more robust and comprehensive AD prediction methodology, enabling better identification and management of individuals at risk of AD and related conditions.


(8) $$\begin{array}{l} P=\frac{\sum \left(y-ȳ\right)\left(ŷ-ȳ\right)}{\sqrt{\sum {\left(y-ȳ\right)}^{2}{\left(ŷ-ȳ\right)}^{2}}} \end{array}$$

where *y* is the predicted EEG signal for the subject in different frequency bands and time periods. *P* represents the prediction result of the EEG signal for the subject. When the value tends toward 1, it indicates that the EEG signal prediction aligns with the MMSE assessment result, validating the credibility of the EEG prediction. Conversely, when the value is close to 0, the EEG prediction is considered less credible, as it does not align well with the MMSE assessment. This correlation analysis provides a quantitative measure to assess the consistency between the predicted EEG signal results and the MMSE, helping to ascertain the reliability of our prediction model.

Based on the Pearson's correlation coefficient, this paper proposes the following formula, which combines the predicted results of the EEG signal with the results of the MMSE scale assessment to obtain the combined prediction.


(9) $$\begin{array}{l} AD_{s}core=\text{λ}\times avg\left(\sum_{i=1}^{\text{y}}EEG\right)+\\ \;(1-\text{λ})\times avg\left(MMSE_{s}core\right) \end{array}$$

where *AD*_*s*_*core* represents the EEG signal prediction results and the assessment results of the MMSE, as shown in Table 1. It is the combined prediction result of EEG signal prediction result and the MMSE assessment after correlation analysis.

**Table 1:** MMSE scoring metrics.

**Methods**	**AD**	**FTD**	**NC**
MMSE	27	10–27	<10
MMSE_score	2	1	0

**Table 2:** The AD dataset is divided into training and test sets.

**Datasets**	**Category label**	**Case**	**Year (Mean ± SD)**	**NPI-Q (Mean ± SD)**
	AD	8/16	70.3 ± 5.5	23.3 ± 2.0
Train (60)	FTD	10/6	64.9 ± 6.6	27.3 ± 1.8
	AD	13/7	65.8 ± 4.0	29.1 ± 1.0
	AD	4/8	64.2 ± 6.0	23.2 ± 2.2
Test (28)	FTD	4/3	61.7 ± 6.6	28.3 ± 1.6
	NC	5/4	64.8 ± 5.8	26.6 ± 1.7

In the above formula, EEG prediction results and MMSE assessment results are controlled by parameters. The formulas are as follows.


(10) $$\begin{array}{l} \gamma =P \end{array}$$

where γ is the Pearson correlation coefficient of the EEG signal prediction result. At that time, the algorithm will only take EEG signal prediction results. At that time, the algorithm will focus on the MMSE scale assessment, subject to clinician assessment.

## 4. Experiments

In this section, we experimentally validate and evaluate the performance of the proposed AD prediction approach.

### 4.1. Dataset acquisition and preprocessing

This study utilized a publicly available dataset containing scalp EEG recordings of individuals with Alzheimer's disease, which can be accessed at the following link: 10.18112/openneuro.ds004504.v1.0.2. EEG signals were acquired using 19 Ag/AgCl electrodes, following the standardized 10–20 international lead systems. The recordings were conducted at a sampling rate of 250 Hz, with a resolution of 10 uV/mm.

The dataset consisted of a total of 88 subjects, divided into three groups: 36 subjects in the AD group, 23 subjects in the FTD group, and 29 subjects in the NC group. The cognitive and neuropsychological status of the subjects was assessed using the well-established MMSE. Ethical considerations were meticulously followed, and the study received approval from the Scientific and Ethical Committee of the Aristotle University of Thessaloniki and AHEPA University Hospital, under protocol number 142/12-04-2023. The availability of the dataset and adherence to ethical guidelines underscore the credibility and reliability of the data used in this study for predicting AD based on EEG signals.

**Figure 3:**
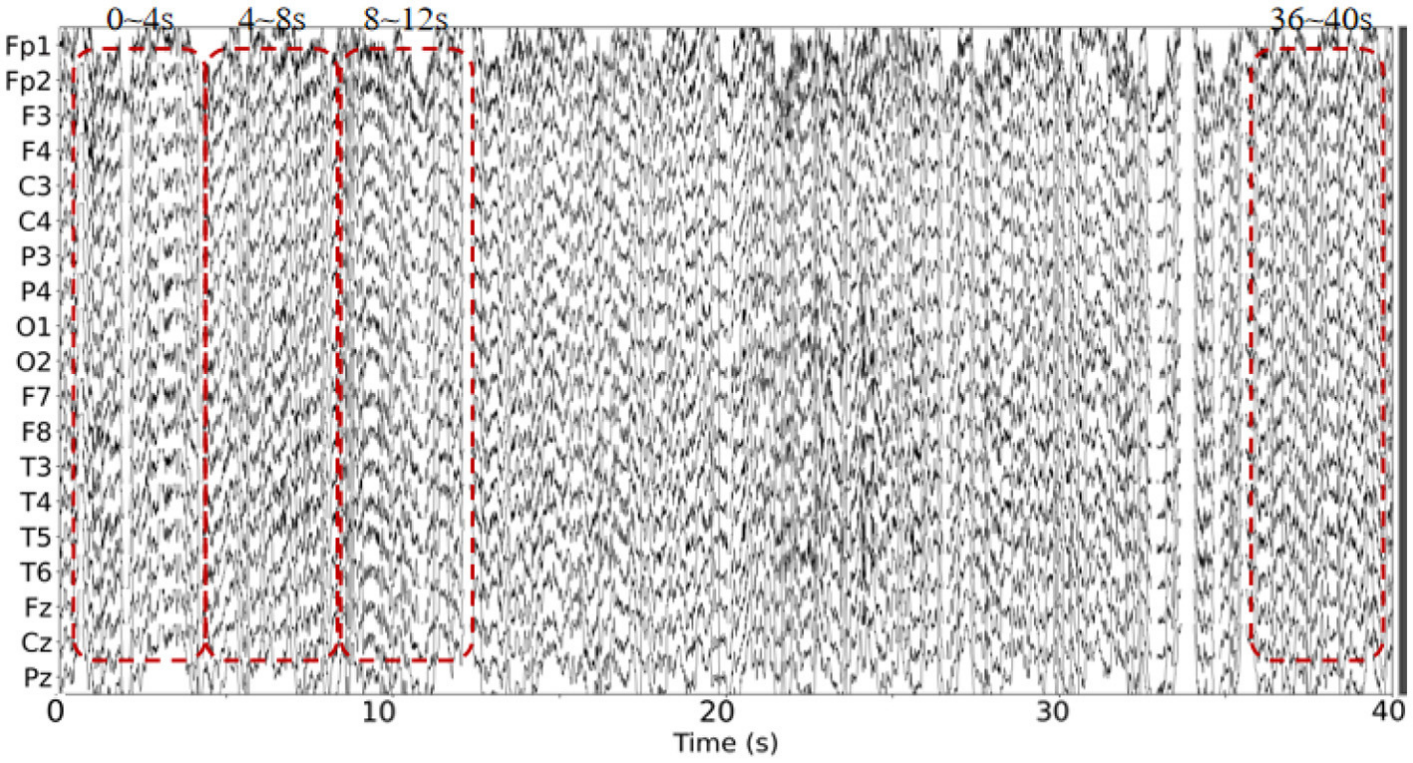
The EEG signal is extracted in the time domain.

**Figure 4:**
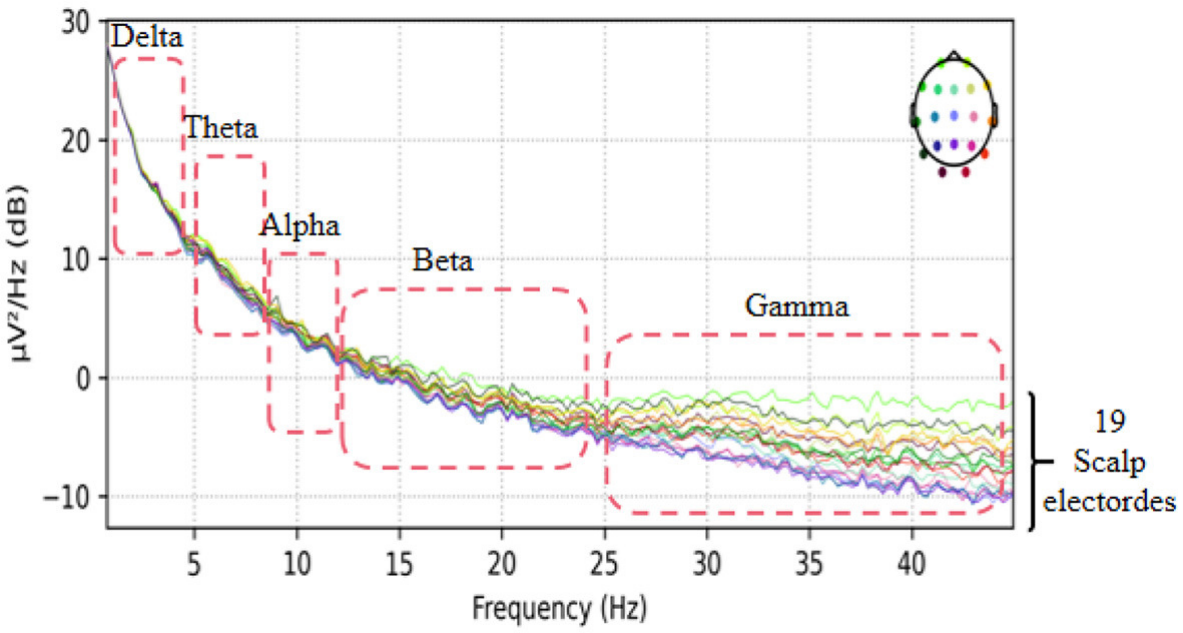
The EEG signal is extracted in the frequency domain.

This dataset has done the preprocessing work such as denoising and removing artifacts after collecting the information of the subjects, so the method in this paper directly uses the data in this dataset without repeating the data preprocessing. The EEG data extracted in this paper are divided into two groups, as follows: (1) As shown in Figure 3, we extracted a total of 10 time segments (4s a segment, the signal of one electrode channel in each segment); (2) As shown in Figure 4, we extracted Delta wave (0.5–4 Hz), Theta wave (4–8 Hz) for each subject, Alpha wave (8–13 Hz), Beta wave (13–25 Hz) and Gamma wave (25–45 Hz) for each subject.

### 4.2. Experimental environment and training strategy

The model was trained and tested on a Windows operating system. The training learning rate was set to 0.0005, and the maximum number of epochs was limited to 100. A batch size of four was used during the training process. Additionally, ten-fold cross-validation was employed to enhance the robustness and generalization of the model's performance.

### 4.3. Evaluation metrics

There is sensitivity (*TP*), which is the probability that the subject will be diagnosed with the disease. Specificity *TN*), which is the probability that the subject will be correctly diagnosed. And 1-specificity is (*FP*), that is, the probability of misdiagnosis. Clinically, we want *TP* as high as possible and *FP* as low as possible. Accuracy, sensitivity, and specificity are calculated as follows:


(11) $$\begin{array}{l} Accuracy\left(in\%\right)=\frac{TP+TN}{TP+TN+FP+FN}\times 100 \end{array}$$

(12) $$\begin{array}{l} Sensitivity\left(in\%\right)=\frac{TP}{TP+FN}\times 100 \end{array}$$

(13) $$\begin{array}{l} 1-Specificity\left(in\%\right)=1-\frac{TN}{TN+FP}\times 100 \end{array}$$

In general, prediction models with high sensitivity and specificity values will be more suitable for AD prediction. The ROC (Receiver Operating Characteristics) curve can clearly show the classification performance of different models. In addition, the area composed of ROC curve and horizontal coordinate (AUC) is also an important index to express the classification performance of the model. The AUC formula is expressed as follows:


(14) $$\begin{array}{l} AUC=\frac{1}{2}\overset{n-1}{\underset{i=1}{\sum }}\left(x_{i-1}-x_{i}\right)\left(y_{i}+y_{i+1}\right) \end{array}$$

where *x* represents the 1-specificity value, *y* represents the sensitivity value

### 4.4. Experimental results and analysis

**Figure 5:**
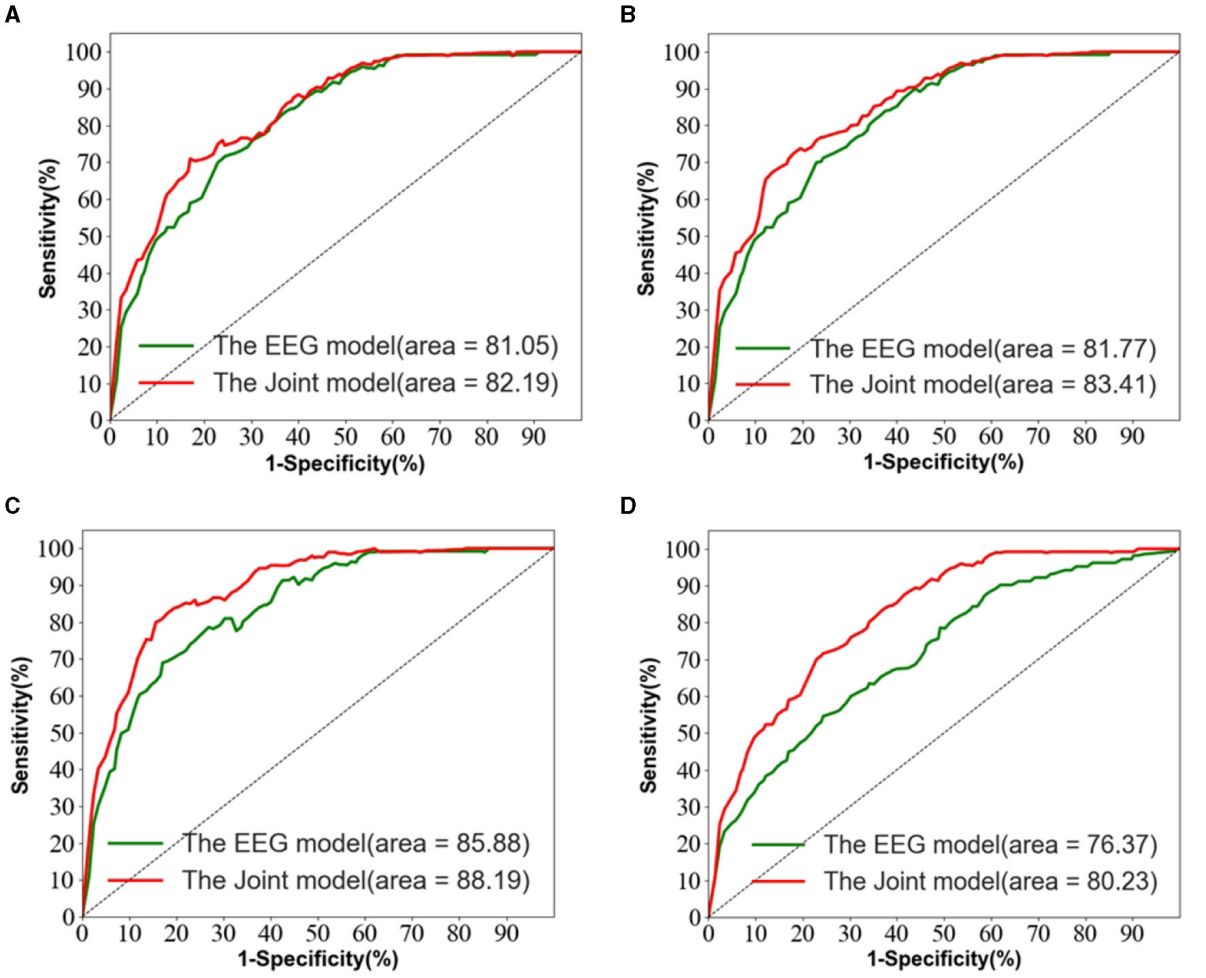
The EEG signal is extracted in the frequency domain. **(A)** AD vs. MCI classification ROC curve. **(B)** MCI vs. NC classification ROC curve. **(C)** AD vs. NC classification curve. **(D)** AD vs. MCI vs. NC classification ROC curve.

Figure 5 depicts the ROC curves for AD vs. NC, FTD vs. NC, AD vs. FTD, and AD vs. FTD vs. NC tasks on the test set. The results indicate that the joint prediction method proposed in this study exhibits superior performance and achieves a higher AUC value compared to the AD prediction method solely based on EEG signals. This improvement can be attributed to the joint diagnosis method's alignment with the clinician's diagnosis, rendering it more effective in AD prediction.

However, it is worth noting that in Figure 5D, the ROC curves of the joint prediction approach closely resemble those of the unimodal model when the one-specificity is at 20% for the AD vs. FTD and FTD vs. NC tasks. This similarity can be attributed to the influence of the MMSE scale, which may be impacted by the subjects' educational backgrounds. Consequently, this sensitivity to educational factors may somewhat restrict the classification performance of the joint prediction method in these specific scenarios.

Table 3 presents a comparison of the two methods proposed in this study with state-of-the-art prediction methods in AD vs. FTD, FTD vs. NC, AD vs. NC, and AD vs. FTD vs. NC classification tasks. These methods include machine learning-based AD prediction methods using EEG signals, where the LightGBM and Random Forests methods exhibit higher AD prediction accuracy. The machine learning-based methods demonstrate good AD prediction accuracy but have notable drawbacks. EEG signals acquired clinically often contain substantial noisy information, requiring complex data preprocessing, posing challenges in clinical research. Therefore, machine learning-based AD prediction methods using EEG signals have limitations.

**Table 3:** Comparison of this paper's method with other methods using EEG signals.

**Method**	**Subjects**			**Data**	**Classification results (%)**				
	**AD**	**FTD**	**NC**	**Type**	**Base**	**SEN**	**SPE**	**ACC**	**AUC**
LightGBM	36	–	29	EEG	OpenNeure	76.01	76.16	76.43	–
SVM	36	–	29	EEG	OpenNeure	71.89	75.98	76.43	–
KNN	36	–	29	EEG	OpenNeure	69.67	74.19	71.23	–
MLP	36	–	29	EEG	OpenNeure	73.00	74.63	73.12	–
Random forests	36	–	29	EEG	OpenNeure	78.32	80.94	77.01	–
EEG model	36	–	29	EEG	OpenNeure	81.76	83.22	85.78	85.88
The joint method	36	–	29	EEG+MMSE	OpenNeure	**84.56**	**85.15**	**87.33**	**88.19**
LightGBM	–	23	29	EEG	OpenNeure	61.13	80.74	72.43	–
SVM	–	23	29	EEG	OpenNeure	62.41	75.98	70.14	–
KNN	–	23	29	EEG	OpenNeure	59.67	76.13	67.34	–
MLP	–	23	29	EEG	OpenNeure	63.00	78.63	73.12	–
Random forests	–	23	29	EEG	OpenNeure	72.32	80.94	72.01	–
EEG model	–	23	29	EEG	OpenNeure	76.34	79.77	80.36	81.77
The joint method	–	23	29	EEG+MMSE	OpenNeure	**81.66**	**82.55**	**82.98**	**83.41**
EEG model	36	23	–	EEG	OpenNeure	77.47	80.12	79.03	81.05
The joint method	36	23	–	EEG+MMSE	OpenNeure	**79.81**	**80.77**	**81.56**	**82.19**
EEG model	36	23	29	EEG	OpenNeure	73.20	75.45	76.01	76.37
The joint method	36	23	29	EEG+MMSE	OpenNeure	**77.09**	**78.30**	**79.12**	**80.23**

Bold values signifies that the metrics reported in this paper exhibit higher values in comparison to those of alternative methods.

In contrast, deep learning methods outperform traditional machine learning methods due to their lower data preprocessing requirements and the ability to extract frequency pattern information from EEG signals, facilitating higher accuracy in AD prediction. The method proposed in this paper effectively predicts the AD class and distinguishes the NC class, with ACC values in the more challenging AD vs. FTD and FTD vs. NC categorizations being over 3% higher than traditional machine learning methods. This improvement is attributed to the deep learning method's capacity to extract information differences between classes.

In the context of addressing the AD vs. NC classification problem, we conducted a comprehensive performance evaluation by comparing our method with other contemporary ensemble classifiers, namely Multilayer Perceptron (MLP), Random Forests, and LightGBM. Our findings reveal a substantial superiority of our method, notably surpassing the second-ranking Random Forests by an impressive margin of 10.32% in terms of accuracy. Furthermore, to provide a well-rounded assessment, we extended our evaluation to the FTD vs. NC classification problem. By applying the same algorithms, we established that our method consistently outperformed the alternatives. Specifically, in terms of accuracy, our approach outshone the second-ranking MLP by 9.86%. Finally, the complexity of AD vs. FTD vs. NC classification was addressed, yielding accuracy, and AUC values of 79.12 and 80.23%, respectively. Nevertheless, it is important to acknowledge that the classification performance in this scenario did not attain remarkable levels. One plausible explanation for this diminished performance could be attributed to the inherent challenge of effectively capturing pertinent information within the data through the proposed joint approach. This limitation, in turn, contributes to the observed lower classification accuracy. It is worth considering that the suboptimal performance might be mitigated with a more substantial training dataset. As the size of the training dataset expands, it is conceivable that the method presented in this study may exhibit improved performance, thereby yielding more promising results.

The proposed method in this paper integrates deep learning algorithms, considering both global features extracted using ViT to address noisy electrode signals in the time domain and weak inter-class signal differences extracted using CNN in the frequency domain. Additionally, a feature filtering module is constructed after fusing global and local features, which emphasizes essential feature signals and reduces redundant information in the feature vector, facilitating the extraction of AD lesion signals.

Furthermore, multimodal-based disease prediction methods gain significant interest due to the need to combine multiple approaches effectively for better clinical prediction results. Relying on a single method may lead to misdiagnosis due to various factors, including sampling data noise interference and physiological heterogeneity among subjects. In this study, we found that matching the MMSE assessment scale scoring values with the EEG signal data of the subjects significantly contributes to the high prediction accuracy.

We also observe from Table 3 that in the FTD vs. NC case, the joint diagnostic algorithm achieves an AUC value of 83.41%, which is superior to the state-of-the-art technology in terms of AUC value. However, it can be seen that a single EEG signal prediction modeling algorithm is no worse than a combined diagnostic algorithm in terms of sensitivity and specificity. This is because the proposed method (joint) incorporates neuropsychological diagnostic information such as MMSE. however, MMSE is influenced by the level of education of the individual. For example, FTD patients with a high level of education have similar scores on the MMSE test as NC patients with a low level of education. In such cases, it is difficult to distinguish between FTD and NC.

The results in Table 3 demonstrate that the AD prediction model and the joint prediction algorithm proposed in this study using only EEG signals achieve higher prediction accuracies compared to other AD prediction methods. The AUC values of the joint prediction in the more challenging AD vs. FTD and FTD vs. NC categorizations reach 82.19 and 83.41%, respectively. These findings highlight the ability of the AD prediction method proposed in this paper to capture AD lesion signals and provide reliable information for AD clinical risk assessment.

In the AD vs. FTD vs. NC triple classification task, the combined prediction algorithm achieves an ACC value of 80.23%, which is 3.86% higher than using only EEG signals (76.37%). This indicates that the combination of EEG signal prediction results with clinical psychology assessment results effectively differentiates between AD, FTD, and NC categories, offering reliable auxiliary diagnostic information for early AD screening.

## 5. The interpretation and visualization of the model (EEG signal only)

### 5.1. The model classification performance analysis

**Figure 6:**
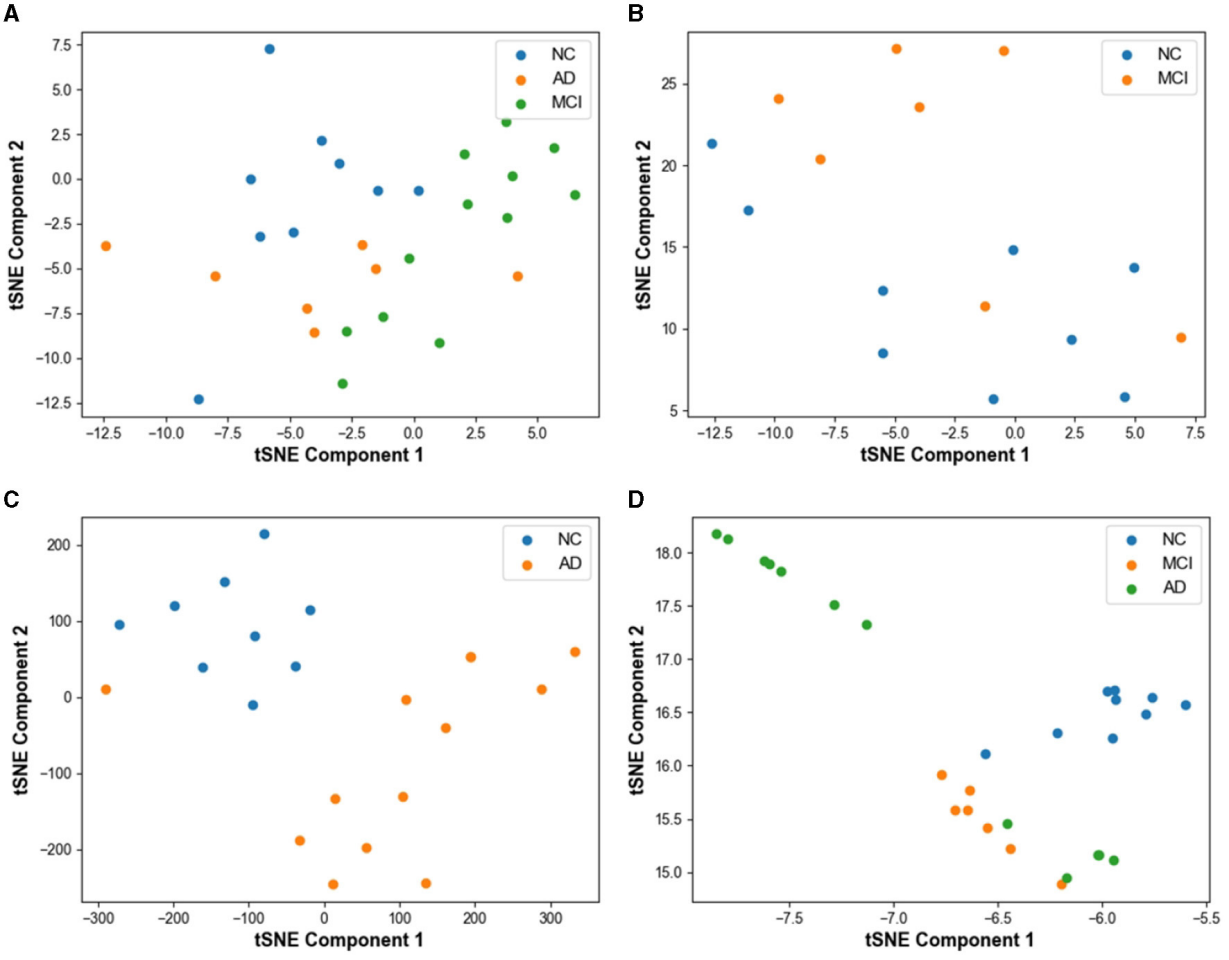
Visualization of model prediction results using t-SNE based tool on test set. **(A)** Result without classification. **(B)** MCI vs. NC classification result with t-SNE. **(C)** AD vs. NC classification result with t-SNE. **(D)** AD vs. MCI vs. NC classification result with t-SNE.

Figure 6 displays the classification performance visualization of the AD prediction model based on EEG signals using the t-SNE tool. In Figure 6A, the distribution of samples from 28 subjects in the test set demonstrates a mixed state before model classification. The samples from the three categories exhibit overlapping, indicating that the classes are not clearly distinguishable initially.

In Figure 6B, the feature visualization reveals that the clustering distance between FTD and NC is relatively close, yet the two categories are mostly distinct. However, some mixed data points can be observed on the graph, resulting from the small differences between NC and FTD categories. Clinically, it is often challenging to determine whether an NC subject has FTD or not, given the relatively blurred boundaries between the two conditions.

For the AD vs. NC classification task (Figure 6C), almost all AD and NC samples are located in their respective clusters, indicating the model's high sensitivity to differentiating AD and NC subjects. The model already possesses the capability to effectively differentiate between these categories.

In Figure 6D, representing the AD vs. FTD vs. NC classification task, FTD and NC samples form dense clusters, predominantly distributed within their respective regions, which is acceptable. However, a small number of AD misclassifications among NC samples can be observed. This could be attributed to the heterogeneous differences among subjects and noise contamination during the data acquisition process, leading to some errors in the EEG signal-based prediction.

Overall, the t-SNE tool provides valuable insights into the model's classification performance and the distinguishability of different categories. The model's high sensitivity to AD and NC, along with the relatively clear clustering of FTD and NC, demonstrates the effectiveness of the AD prediction model based on EEG signals.

### 5.2. Visualization of the model

To investigate the electrodes that are highly relevant to the AD prediction model based on EEG signals proposed in this study, we extracted the weights associated with the 19 channels and mapped them onto the brain topography map to gain insights into the model's prediction process.

**Figure 7:**
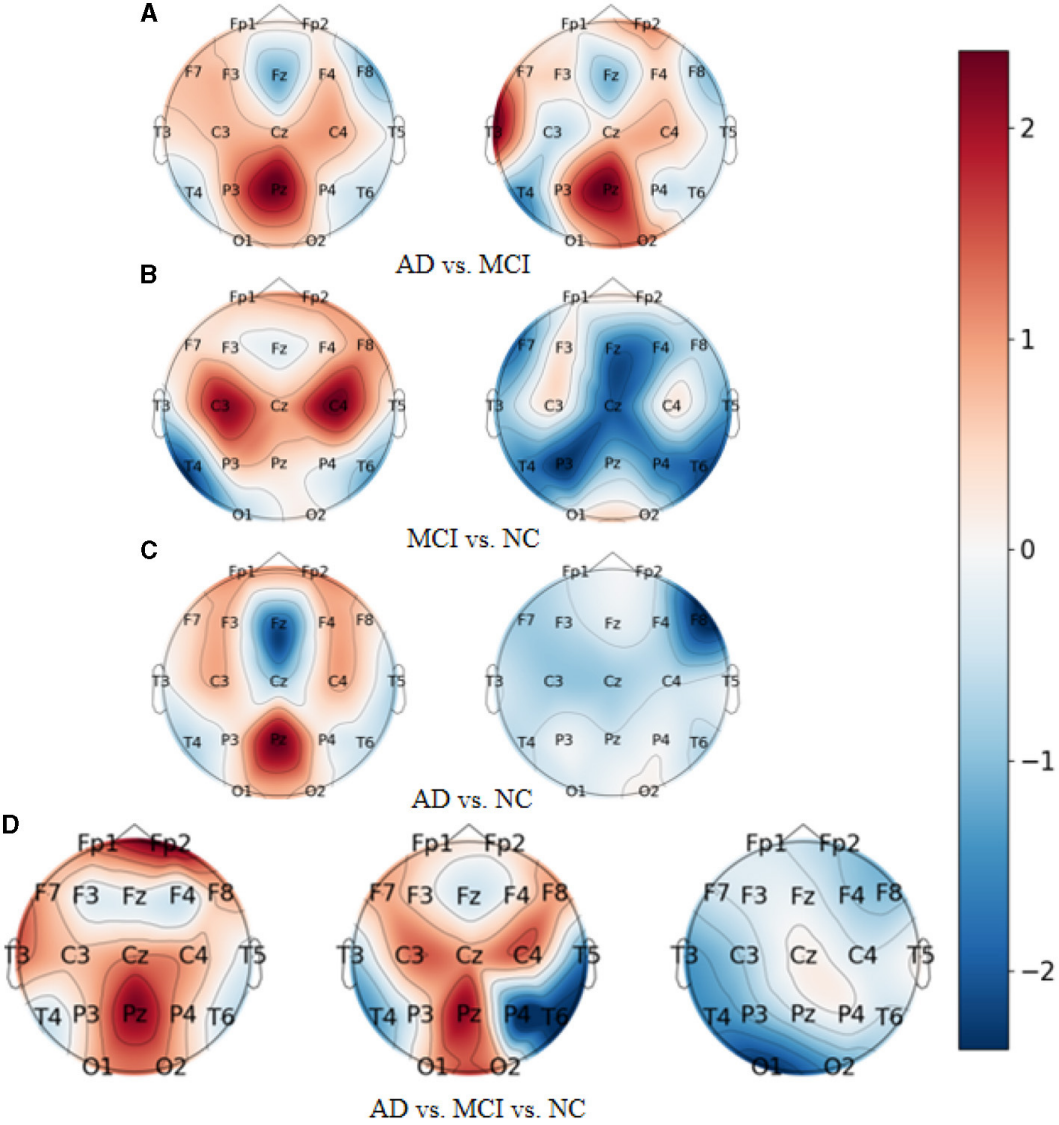
Topography of model visualization under different prediction tasks. **(A)** AD vs. MCI. **(B)** MCI vs. NC. **(C)** AD vs. NC. **(D)** AD vs. MCI vs. NC.

As demonstrated in Figures 7A–D, which represent the topographic maps of EEG electrode weights under the four categorization tasks of AD vs. FTD, FTD vs. NC, AD vs. NC, and AD vs. FTD vs. NC, respectively, we can observe the following:

For AD patients, the model primarily focuses on AD-related features in the frontal lobe (Fp1 and Fp2 electrodes), parietal lobe (P3, Pz, and P4 electrodes), and occipital lobe (O1 and O2 electrodes). These regions correspond to the frontal lobe, parietal temporal region, hippocampus, internal olfactory cortex, posterior cingulate sulcus, and occipital lobe, which is consistent with clinical studies on AD diseases (Kim et al., 2020).

For FTD patients, the model shows greater sensitivity toward the central region (C3, Cz, and C4 electrodes), the left temporal lobe (T3 electrode), and the frontal lobe (Fp1, Fp2, F7, and F8 electrodes). This increased focus on the temporal lobe and central region aligns with clinical neurological findings related to FTD disorders (Ding et al., 2019).

For NC subjects, the model displays higher sensitivity toward the occipital lobe (O1 and O2 electrodes), the temporal lobe (T3 and T5 electrodes), and the left and right parietal lobes (C3 and C4 electrodes). These areas are known to be associated with lesion occurrences (Amira et al., 2014).

Overall, the model's electrode weight mappings reflect its focus on specific brain regions corresponding to AD, FTD, and NC conditions, and these findings are consistent with established clinical studies. The visualization of electrode weight distributions enhances our understanding of the model's prediction process and the important features it utilizes for accurate AD classification and risk assessment.

**Figure 8:**
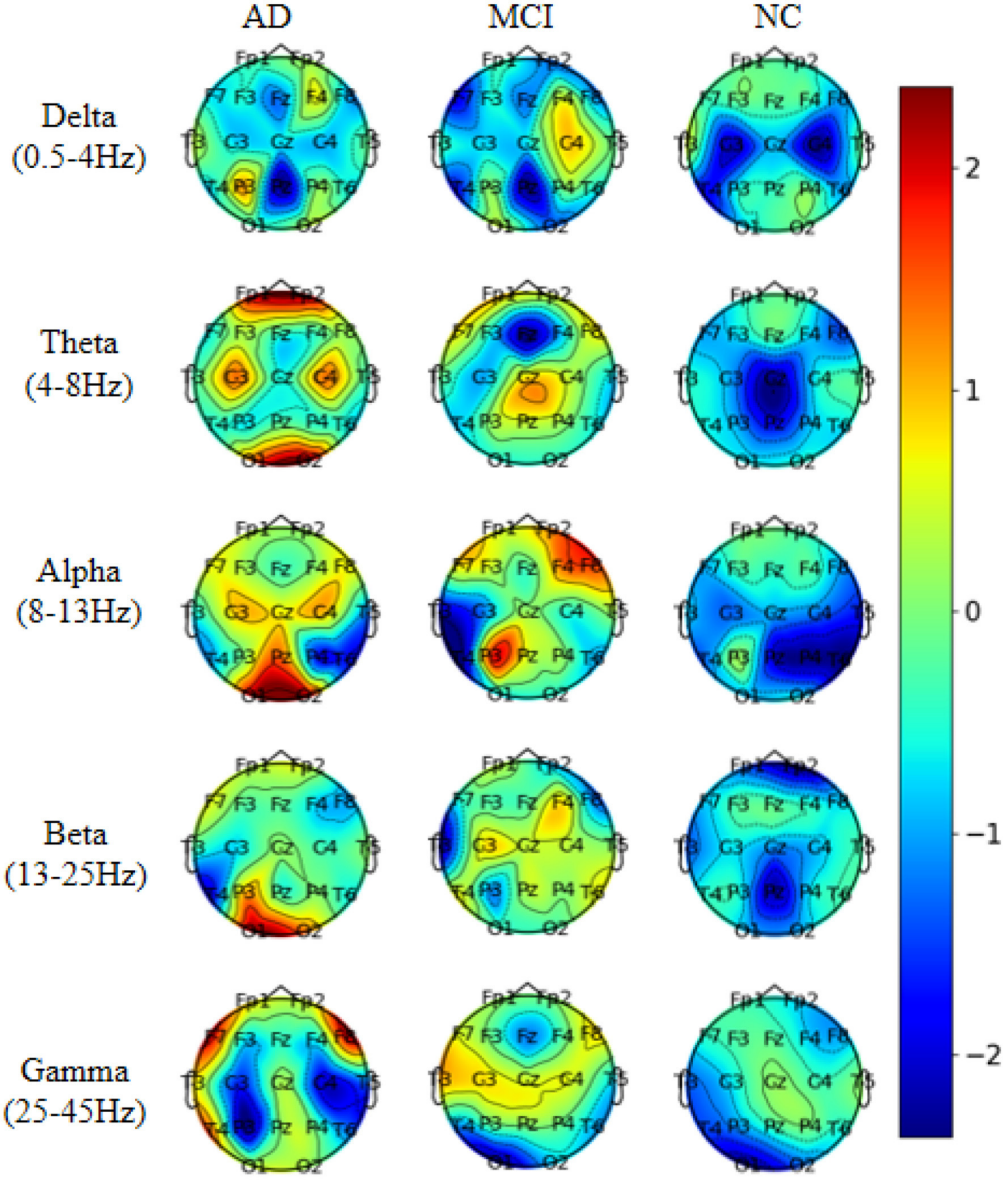
Visualization of topographic maps in different frequency bands.

To investigate the electrodes used in different frequency bands in the model, we conducted experiments on the AD vs. FTD vs. NC classification task. We extracted five frequency bands for each EEG signal and mapped the weights of the 19 electrode channels in each frequency band onto a brain topographic map (Figure 8).

From Figure 8, we observed that Delta (0.5–4 Hz) waves showed minimal differences between AD and FTD, suggesting that Delta waves may not carry significant focal information in the pathogenesis of AD. Conversely, Theta (4–8 Hz) and Alpha (8–13 Hz) waves exhibited more pronounced differences on topographic maps of AD, FTD, and NC. The weight distributions reflected deeper involvement of the occipital, temporal, and frontal lobes, which are commonly implicated regions in the pathogenesis of AD. Additionally, Beta (13–25 Hz) and Gamma (25–45 Hz) waves also displayed substantial differences in AD and FTD cases, with heavier weighting and predominant distribution in the occipital and frontal regions, aligning with clinical findings (Miltiadous et al., 2023b).

These results shed light on the distinctive contributions of different frequency bands in the model's classification task. Theta and Alpha waves appear to play a crucial role in distinguishing between AD, FTD, and NC subjects, as they highlight significant differences in brain regions associated with AD pathology. On the other hand, Delta waves seem less discriminative, while Beta and Gamma waves also contribute to differentiating AD and FTD cases, particularly in the occipital and frontal regions. This analysis enhances our understanding of the model's utilization of frequency-specific information for accurate classification and risk assessment of AD and related conditions.

### 5.3. Ablation experiments

The accuracy of the joint prediction method proposed in this study is contingent upon both the prediction accuracy of the EEG signal and the accuracy of the MMSE evaluation. As the MMSE score follows a standardized assessment, we conducted ablation experiments on the EEG signal-based AD prediction model to validate the state-of-the-art performance of the model proposed in this paper. These ablation experiments were specifically carried out on the AD vs. FTD vs. NC classification task, providing a rigorous assessment of the model's overall performance and its contribution to the joint prediction approach.

#### 5.3.1. Experiments on ablation of feature fusion strategies for two-branch networks

To verify the advancement of the feature fusion strategy of the two-branch network proposed in this paper. The results of this ablation experiment are shown in Table 4.

**Table 4:** Ablation experiments of the proposed feature fusion strategy for two-branch networks.

**Methods**	**AD vs. FTD vs. NC (%)**			
	**SEN**	**SPE**	**ACC**	**AUC**
Single-branch model (CNN)	54.25	57.48	57.56	58.97
Single-branch model (ViT)	62.47	64.88	65.33	66.61
Double-branch model (CNN-CNN)	65.83	67.26	66.89	68.33
Double-branch model (ViT-ViT)	70.56	72.25	74.79	74.12
Ours(ViT-CNN)	**73.20**	**75.45**	**76.01**	**76.37**

Bold values signifies that the metrics reported in this paper exhibit higher values in comparison to those of alternative methods.

We conducted ablation experiments on the network structure while keeping other parameters unchanged and validated the results on the test set.

In Table 4, we observed that the ACC and AUC values of the dual-branch model proposed in this paper are the highest, outperforming the dual-branch CNN model and the dual-branch ViT model. This suggests that our proposed method efficiently captures both the texture features and the overall information distribution features in the EEG signal. The dual-branch structure exhibits better feature extraction capabilities compared to the single-branch structure, mainly due to its ability to mitigate feature signal loss during the information acquisition process from the EEG signal.

**Figure 9:**
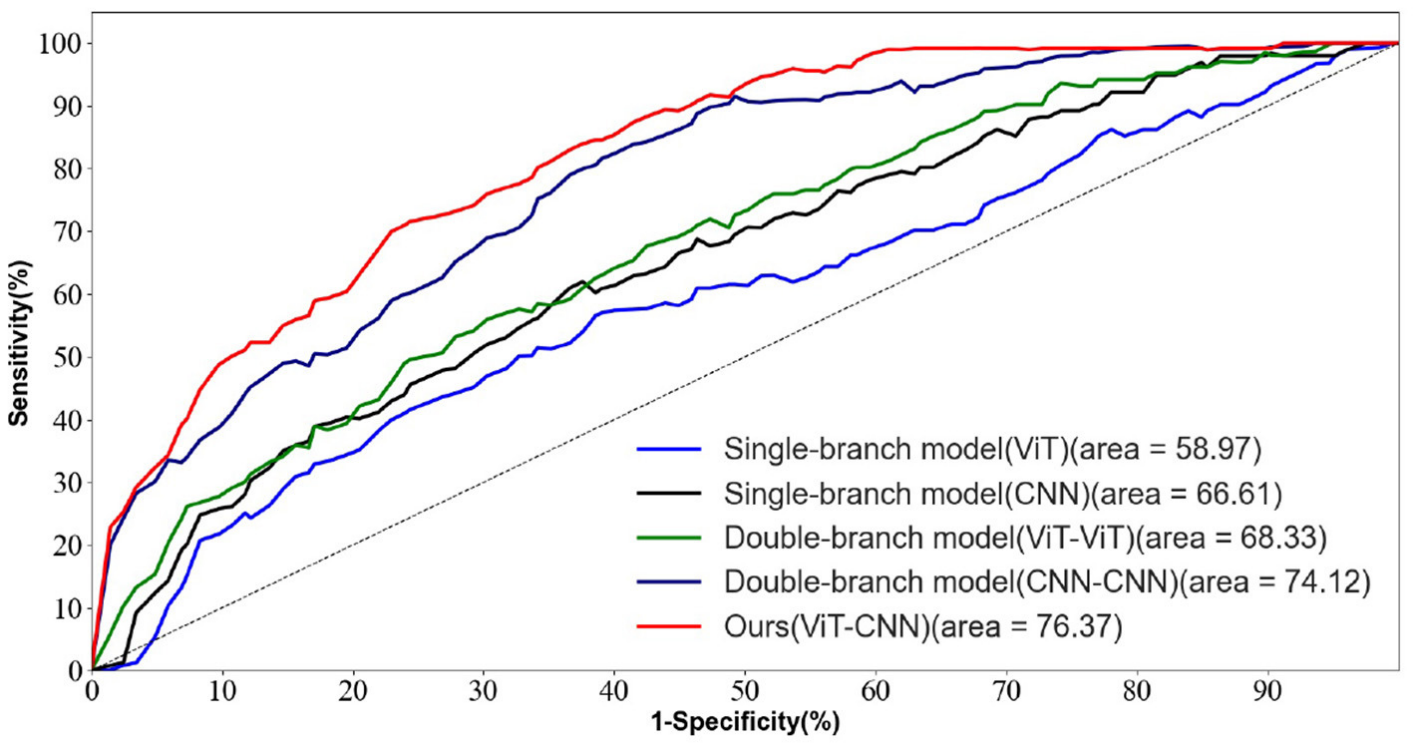
ROC curves for different network structures in the AD vs. FTD vs. NC task.

As shown in Figure 9, the ROC curve of the two-branch model (CNN-ViT) proposed in this paper is smooth and maintains high sensitivity while achieving high specificity. This indicates that our method achieves a good balance between sensitivity and specificity in AD prediction.

Overall, the ablation experiments and ROC curve analysis further validate the effectiveness and superiority of the dual-branch model proposed in this paper for AD prediction based on EEG signals.

#### 5.3.2. Feature screening module ablation experiments

To validate the advancement of the feature screening module proposed in this paper. The results of this ablation experiment are shown in Table 5.

**Table 5:** Ablation experiments with different loss functions.

**Methods**	**AD vs. FTD vs. NC (%)**			
	**SEN**	**SPE**	**ACC**	**AUC**
Not-feature filter	69.58	71.14	72.23	73.15
Feature filter	**73.20**	**75.45**	**76.01**	**76.37**

Bold values signifies that the metrics reported in this paper exhibit higher values in comparison to those of alternative methods.

We trained the AD prediction model without the feature screening module using EEG signals while keeping other parameters unchanged. As shown in Table 5, the ACC value of the model using the feature screening module is higher than the ACC value of the model without the feature screening module. This indicates that the feature screening module plays a crucial role in learning AD lesion signal features and suppressing noise information.

**Figure 10:**
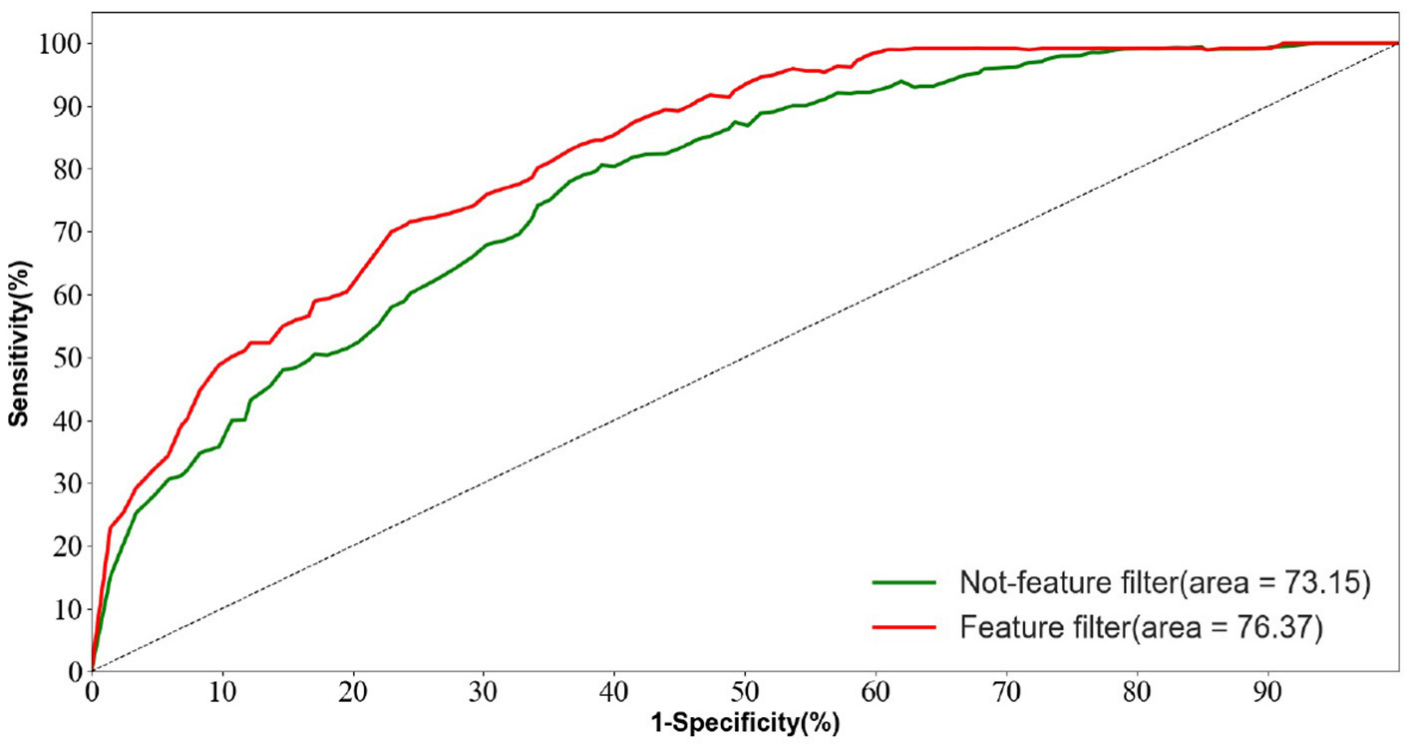
Feature screening module for ablation experiments.

Figure 10 displays the ROC curves of the two groups of prediction models. It can be observed that the ROC curves of the models with feature screening are smoother than those of the models without feature screening. Additionally, the AUC values of the models with feature screening are higher than those of the models without feature screening when the 1-specificity value is between 30 and 50%. This finding suggests that the feature screening module exhibits stronger sensitivity to FTD and AD feature signals. In clinical neurological disease diagnosis, high sensitivity and low 1-specificity are crucial criteria for evaluating the classification performance of prediction networks. Hence, the feature screening module significantly enhances the classification performance of the model.

### 5.4. Discussion

This study introduces an innovative deep learning architecture based on multi-feature fusion learning, tailored for the discrimination of clinical dementia using EEG signals. Specifically, the method is devised for the detection of AD, while also aiming to evaluate its potential applicability to other forms of dementia. The methodology comprises three distinct phases.

In the initial phase, raw EEG signals, diligently collected from the neurology department, undergo rigorous processing, resulting in the segmentation of a 40 s time window into ~10 segments. The ensuing phase involves the transformation of EEG signals into the time-frequency domain. To achieve this, the Welch method, a frequency-domain transformation technique for EEG signals, is employed. This method employs a sliding window approach in conjunction with fast Fourier transform to derive frequency-domain signals. In the final step, a deep neural network is trained, which is comprised of two parallel CNNs alongside a transformer network. The training process involves multiple iterations, optimizing model performance through the utilization of cross-entropy loss.

From a medical perspective, the proposition of a novel multi-feature fusion learning architecture, leveraging ViT and CNNs, for the classification of AD within EEG signals holds substantial significance. Automated early AD detection with minimal medical intervention is pivotal for timely treatment and management. Notably, EEG signals have emerged as crucial tools in neurological disorder diagnosis. Although positron emission tomography (PET) and magnetic resonance imaging (MRI) are prevalent imaging modalities for AD detection, EEG provides a swifter, cost-effective, and portable alternative.

The proposed architecture adeptly captures prominent EEG changes associated with AD, including reduced alpha and beta waves, diminished cortical activity, increased theta waves, and decreased inter-brain region synchronization. The potential to identify these changes in early-stage disease through a machine learning framework positions EEG as a viable biomarker for AD. However, it is noteworthy that the model exhibited limited capacity to effectively discriminate between FTD and NC classes, aligning with existing clinical research outcomes. Moreover, the training of an efficient ViT weight for AD prediction necessitated a substantial volume of data, revealing the dearth of extensive research in the realm of AD prediction using EEG signals. As such, the findings of this paper underscore the necessity for validation through extensive clinical data to affirm the method's applicability and potential in the field.

## 6. Conclusion

The research paper introduces a novel and innovative methodology that integrates EEG signals with clinical psychological scale assessment to collectively predict AD. The pivotal contribution of our work is the introduction of a two-branch network architecture, comprising CNN and ViT. This strategic amalgamation effectively harnesses the unique strengths of each component, enabling the model to extract essential features from EEG electrode signals and diverse frequency bands. The resulting enriched feature representation facilitates the detection of focal features intricately associated with AD. Further enhancement through a feature screening module empowers the model to highlight pertinent AD-related signal features while suppressing extraneous noise, thereby augmenting the predictive power of the model. Empirical validation substantiates the superiority of our proposed methodology over conventional AD prediction techniques that solely rely on EEG signals. Notably, our approach surpasses existing methods in terms of both Overall ACC and the AUC, achieving notable ACC rates of 81.56 and 82.98%, respectively. Moreover, the integration of visualization experiments lends further credibility to our model's feature extraction process.

In the context of forthcoming research endeavors, it is advisable to give due consideration to augmenting the datasets employed for both model training and testing. This augmentation stands as a pivotal endeavor, as the expanded dataset serves a dual role: enhancing the model's robustness while concurrently broadening its sphere of applicability. Simultaneously, it is pertinent to note that the feature screening module, delineated as the attention model in this study, necessitates heightened computational resources. This module, designed to scrutinize fused features and enhance lesion signal attributes while diminishing noisy information, presents a computational complexity surpassing that of conventional attention models. This disparity can lead to challenges such as convergence difficulties and runtime errors, especially when addressing substantial datasets with limited computational resources. Furthermore, a noteworthy trajectory for potential enhancement lies in extending the proposed approach to encompass a comprehensive exploration of other forms of dementia, including FTD and Lewy body dementia. This strategic expansion to encompass distinct dementia types harbors the capacity to furnish a comprehensive evaluation of the diagnostic efficacy of the method within a broader spectrum of neurodegenerative conditions.

In conclusion, the integration of EEG signals and clinical psychological assessments within a unified prediction framework holds substantial promise for advancing the early identification of Alzheimer's Disease. The method's exceptional performance across intricate differentiations underscores its potential as a valuable tool in both clinical and research settings. As the landscape of neurodegenerative disorders evolves, we anticipate that our innovative approach will contribute significantly to improved diagnostic accuracy and ultimately to the development of more effective strategies for managing AD. Nonetheless, we are committed to continual improvement and envision a future where our approach aids healthcare professionals in the precise prediction and management of AD, unlocking the potential for early intervention and improved patient outcomes.

## Data availability statement

Publicly available datasets were analyzed in this study. This data can be found at: Dataset: 10.18112/openneuro.ds004504.v1.0.2. Dataset License: CC0.

## Author contributions

YC: Writing—original draft, Investigation. HW: Writing—review and editing, Formal analysis, Funding acquisition, Methodology, Supervision, Validation. DZ: Writing—review and editing, Data curation, Formal analysis. LZ: Investigation, Validation, Writing—review and editing, Formal analysis. LT: Validation, Writing—review and editing, Methodology, Supervision.
