# Supplementary material for: Multi-feature fusion learning for Alzheimer's disease prediction using EEG signals in resting state
Source: Front Neurosci. 2023 Sep 25;17:1272834. doi: 10.3389/fnins.2023.1272834 (PMC10563817; doi:10.3389/fnins.2023.1272834)
Supplement: Supplementary file 1 [file Data_Sheet_1.PDF]

Supplementary Material

1 SUPPLEMENTARY DATA

No.

2 SUPPLEMENTARY TABLES AND FIGURES

To investigate the electrodes that are highly relevant to the AD prediction model based on EEG signals proposed in this study, we extracted the weights associated with the 19 channels and mapped them onto the brain topography map to gain insights into the model's prediction process. Fig. S1 presents the brain electrode distribution maps before and after obtaining weight mappings from the model output.

In Fig. S1(b), after inputting AD subjects into the model, it is evident that the model pays significant attention to electrode weights mainly distributed in the frontal lobe (Fp1 and Fp2 electrodes), parietal lobe (Pz electrode), and occipital lobe (O1 and O2 electrodes). These findings align with AD lesion areas identified in clinical studies (**?**).

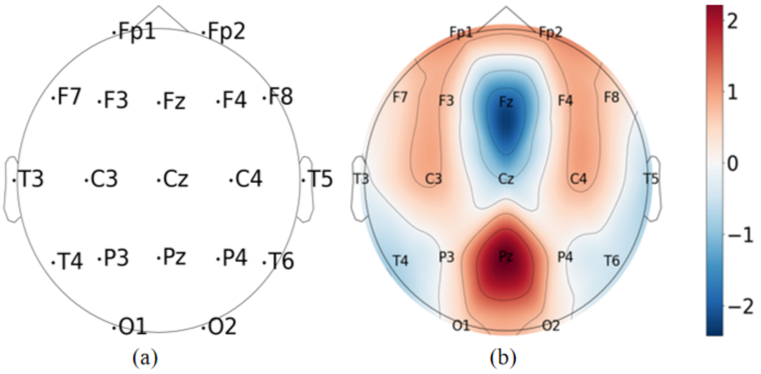

Figure S1. Topography of EEG electrode signals.
